# 1,8-Bis(4-meth­oxy-3-nitro­phen­yl)naphthalene

**DOI:** 10.1107/S1600536811036567

**Published:** 2011-09-14

**Authors:** Panchami Prabhakaran, Vedavati G. Puranik, Gangadhar J. Sanjayan

**Affiliations:** a Division of Organic Chemistry, National Chemical Laboratory, Dr. Homi Bhabha Road, Pune 411 008, India; b Center for Materials Characterization, National Chemical Laboratory, Dr. Homi Bhabha Road, Pune 411 008, India

## Abstract

Mol­ecules of the title compound, C_24_H_18_N_2_O_6_, are located on a twofold rotation axis passing through through the central C—C bond of the naphthalene ring system. The mol­ecular conformation is characterized by a roughly coplanar arrangement of the two substituted phenyl rings [dihedral angle 18.53 (5)°]. These two aryl rings are each twisted by 65.40 (5)° from the plane of the naphthyl unit.

## Related literature

For use of the title compound as a building block for the synthesis of multidentate ligands, see: Sabater *et al.* (2005 ▶); Baruah *et al.* (2007 ▶); Prabhakaran *et al.* (2009 ▶). For the synthesis of the title compound, see: Letsinger *et al.* (1965 ▶); Li *et al.* (2005 ▶).
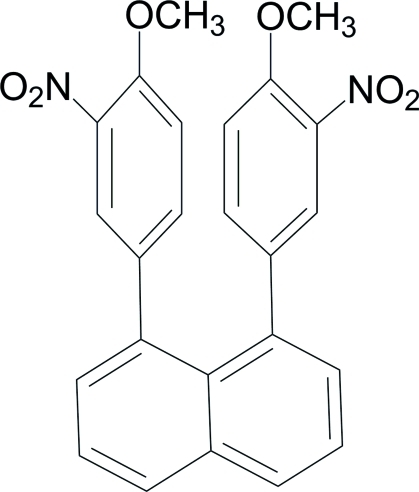


## Experimental

### 

#### Crystal data

C_24_H_18_N_2_O_6_
*M* *_r_* = 430.40
Tetragonal, 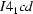

*a* = 13.3038 (9) Å
*c* = 22.7868 (11) Å
*V* = 4033.1 (6) Å^3^
*Z* = 8
Mo *K*α radiation
μ = 0.10 mm^−1^
*T* = 293 K
0.35 × 0.24 × 0.12 mm

#### Data collection

Bruker SMART CCD area-detector diffractometer
Absorption correction: multi-scan (*SADABS*; Bruker, 2003 ▶) *T*_min_ = 0.965, *T*_max_ = 0.988
9753 measured reflections
959 independent reflections
918 reflections with *I* > 2σ(*I*)
*R*_int_ = 0.023

#### Refinement

*R*[*F* ^2^ > 2σ(*F* ^2^)] = 0.035
*wR*(*F* ^2^) = 0.088
*S* = 1.07
959 reflections
156 parameters
1 restraint
Only H-atom displacement parameters refined
Δρ_max_ = 0.19 e Å^−3^
Δρ_min_ = −0.20 e Å^−3^

### 

Data collection: *SMART* (Bruker, 2003 ▶); cell refinement: *SAINT* (Bruker, 2003 ▶); data reduction: *SAINT*; program(s) used to solve structure: *SHELXS97* (Sheldrick, 2008 ▶); program(s) used to refine structure: *SHELXL97* (Sheldrick, 2008 ▶); molecular graphics: *pyMOL* (DeLano, 2004 ▶); software used to prepare material for publication: *SHELXTL* (Sheldrick, 2008 ▶).

## Supplementary Material

Crystal structure: contains datablock(s) I, global. DOI: 10.1107/S1600536811036567/bt5623sup1.cif

Structure factors: contains datablock(s) I. DOI: 10.1107/S1600536811036567/bt5623Isup2.hkl

Supplementary material file. DOI: 10.1107/S1600536811036567/bt5623Isup3.cml

Additional supplementary materials:  crystallographic information; 3D view; checkCIF report

## Figures and Tables

**Fig. 1.**
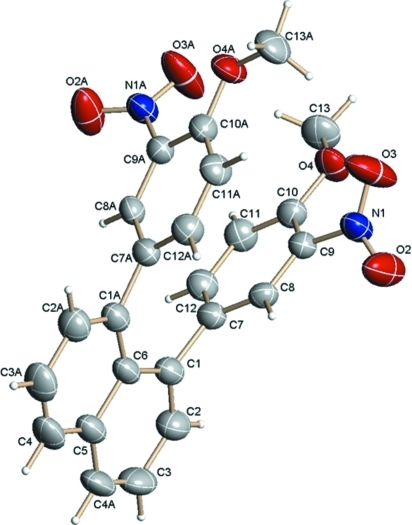
The molecular structure of the title compound, with atom labels and 50% probability displacement ellipsoids for non-H atoms.

## supplementary crystallographic information

### Comment

Rigid building blocks with novel structural features are of considerable interest in designing functional solids. The biaryl based title compound has been synthesized and we report herein its crystal structure. It can be used as a   building block for the synthesis of multidentate ligands (Sabater *et al.*, 2005), foldamer synthesis (Baruah *et al.*, 2007; Prabhakaran *et al.*, 2009) and as a bridging unit in the design of molecules with antiparallel orientation.

The title molecule adopts a 'co-facial' structural architecture (Fig. 1). The two aryl rings are almost parallel orientation to each other and are nearly perpendicular to the rigid naphthyl unit. NO_2_ groups appended on the aryl rings are in *anti* orientation which are in   contrast to the corresponding bis formyl derivative (Sabater *et al.*, 2005).

### Experimental

1,8-naphthalene diboronic acid was synthesized according to the literature procedure (Letsinger *et al.*, 1965). A sealed tube containing 1,8-naphthalene diboronic acid (1 g, 4.6 mmol, 1 equiv.), 4-iodo-1-methoxy-2-nitro benzene (3.87 g, 13.8 mmol, 3 equiv.), DABCO (24 mol%), pottassium carbonate (7 equiv.), TBAB (0.1 equiv.) and Pd(OAc)_2_ (12 mol%) in PEG-400 (4 ml) was subjected to suzuki coupling using a standard procedure (Li *et al.*, 2005). After heating at 110 degree centigrade for 15 h, the tube was broken and the reaction mixture was taken in DCM. The organic layer was washed with dil HCl and the crude product was extracted into the organic layer. Work-up and purification of the crude product by column chromatography afforded an yellow solid (35%). Yellow needle shaped single crystals suitable for X-ray diffraction were obtained by slow evaporation of a solution in a Ethyl acetate -light petroleum ether mixture at room temperature.

### Refinement

All the H atoms were located in a difference Fourier map and refined freely. No atoms heavier than Si were present and a meaningless Flack parameter was obtained, -0.7 (17). Therefore 877 Friedel pairs were merged before final refinement.

### Crystal data

**Table:**

C_24_H_18_N_2_O_6_	*D*_x_ = 1.418 Mg m^−^^3^
*M**_r_* = 430.40	Melting point: 494 K
Tetragonal, *I*4_1_*c**d*	Mo *K*α radiation, λ = 0.71073 Å
Hall symbol: I 4bw -2c	Cell parameters from 4285 reflections
*a* = 13.3038 (9) Å	θ = 2.8–27.5°
*c* = 22.7868 (11) Å	µ = 0.10 mm^−^^1^
*V* = 4033.1 (6) Å^3^	*T* = 293 K
*Z* = 8	Needle, yellow
*F*(000) = 1792	0.35 × 0.24 × 0.12 mm

### Data collection

**Table:**

Bruker SMART CCD area-detector diffractometer	959 independent reflections
Radiation source: fine-focus sealed tube	918 reflections with *I* > 2σ(*I*)
graphite	*R*_int_ = 0.023
ω Scan scans	θ_max_ = 25.5°, θ_min_ = 2.8°
Absorption correction: multi-scan (*SADABS*; Bruker, 2003)	*h* = −12→16
*T*_min_ = 0.965, *T*_max_ = 0.988	*k* = −16→14
9753 measured reflections	*l* = −25→27

### Refinement

**Table:**

Refinement on *F*^2^	Primary atom site location: structure-invariant direct methods
Least-squares matrix: full	Secondary atom site location: difference Fourier map
*R*[*F*^2^ > 2σ(*F*^2^)] = 0.035	Hydrogen site location: inferred from neighbouring sites
*wR*(*F*^2^) = 0.088	Only H-atom displacement parameters refined
*S* = 1.07	*w* = 1/[σ^2^(*F*_o_^2^) + (0.0507*P*)^2^ + 0.8056*P*] where *P* = (*F*_o_^2^ + 2*F*_c_^2^)/3
959 reflections	(Δ/σ)_max_ = 0.001
156 parameters	Δρ_max_ = 0.19 e Å^−^^3^
1 restraint	Δρ_min_ = −0.20 e Å^−^^3^

### Special details

**Table:**

Geometry. All e.s.d.'s (except the e.s.d. in the dihedral angle between two l.s. planes) are estimated using the full covariance matrix. The cell e.s.d.'s are taken into account individually in the estimation of e.s.d.'s in distances, angles and torsion angles; correlations between e.s.d.'s in cell parameters are only used when they are defined by crystal symmetry. An approximate (isotropic) treatment of cell e.s.d.'s is used for estimating e.s.d.'s involving l.s. planes.
Refinement. Refinement of *F*^2^ against ALL reflections. The weighted *R*-factor *wR* and goodness of fit *S* are based on *F*^2^, conventional *R*-factors *R* are based on *F*, with *F* set to zero for negative *F*^2^. The threshold expression of *F*^2^ > σ(*F*^2^) is used only for calculating *R*-factors(gt) *etc*. and is not relevant to the choice of reflections for refinement. *R*-factors based on *F*^2^ are statistically about twice as large as those based on *F*, and *R*- factors based on ALL data will be even larger.

### Fractional atomic coordinates and isotropic or equivalent isotropic displacement parameters (Å^2^)

**Table:**

	*x*	*y*	*z*	*U*_iso_*/*U*_eq_	
N1	0.82311 (12)	0.58354 (11)	0.35152 (7)	0.0469 (4)	
O2	0.76244 (14)	0.63612 (12)	0.32705 (7)	0.0833 (6)	
O3	0.85433 (18)	0.60222 (14)	0.40005 (7)	0.0931 (7)	
O4	0.86899 (11)	0.40341 (10)	0.40686 (6)	0.0546 (4)	
C1	0.92726 (13)	0.43673 (14)	0.16094 (8)	0.0426 (4)	
C2	0.86451 (15)	0.37792 (16)	0.12783 (10)	0.0533 (5)	
H2	0.8188	0.3365	0.1470	0.057 (6)*	
C3	0.86637 (18)	0.37761 (19)	0.06630 (10)	0.0624 (6)	
H3	0.8232	0.3362	0.0452	0.054 (6)*	
C4	1.06781 (17)	0.56135 (18)	0.03803 (9)	0.0603 (6)	
H4	1.0675	0.5600	−0.0028	0.052 (6)*	
C5	1.0000	0.5000	0.06919 (11)	0.0483 (6)	
C6	1.0000	0.5000	0.13242 (11)	0.0416 (5)	
C7	0.91362 (12)	0.42884 (13)	0.22606 (7)	0.0398 (4)	
C8	0.87701 (11)	0.50813 (12)	0.25959 (7)	0.0373 (4)	
H8	0.8617	0.5689	0.2416	0.033 (4)*	
C9	0.86311 (12)	0.49760 (12)	0.31946 (7)	0.0384 (4)	
C10	0.88427 (12)	0.40744 (13)	0.34828 (8)	0.0417 (4)	
C11	0.91814 (14)	0.32758 (13)	0.31419 (9)	0.0460 (4)	
H11	0.9317	0.2660	0.3318	0.056 (5)*	
C12	0.93181 (13)	0.33873 (14)	0.25471 (8)	0.0455 (4)	
H12	0.9540	0.2839	0.2330	0.042 (5)*	
C13	0.89059 (19)	0.31077 (18)	0.43594 (9)	0.0624 (6)	
H13A	0.8490	0.2585	0.4200	0.069 (7)*	
H13B	0.8772	0.3177	0.4771	0.083 (8)*	
H13C	0.9601	0.2938	0.4302	0.067 (6)*	

### Atomic displacement parameters (Å^2^)

**Table:**

	*U*^11^	*U*^22^	*U*^33^	*U*^12^	*U*^13^	*U*^23^
N1	0.0583 (9)	0.0482 (8)	0.0342 (8)	0.0077 (7)	−0.0010 (7)	0.0013 (6)
O2	0.1003 (13)	0.0813 (11)	0.0682 (11)	0.0486 (10)	−0.0270 (10)	−0.0212 (9)
O3	0.1602 (18)	0.0772 (12)	0.0417 (10)	0.0396 (12)	−0.0298 (11)	−0.0138 (8)
O4	0.0720 (9)	0.0572 (8)	0.0347 (7)	0.0103 (6)	0.0072 (6)	0.0128 (6)
C1	0.0463 (9)	0.0471 (9)	0.0345 (9)	0.0074 (7)	−0.0020 (7)	−0.0019 (7)
C2	0.0553 (11)	0.0579 (12)	0.0467 (10)	0.0007 (9)	−0.0051 (10)	−0.0060 (9)
C3	0.0673 (13)	0.0728 (14)	0.0470 (12)	0.0058 (10)	−0.0176 (11)	−0.0178 (10)
C4	0.0719 (13)	0.0799 (15)	0.0292 (9)	0.0164 (12)	0.0086 (9)	0.0108 (10)
C5	0.0563 (15)	0.0583 (15)	0.0305 (14)	0.0153 (11)	0.000	0.000
C6	0.0476 (13)	0.0499 (13)	0.0274 (11)	0.0130 (11)	0.000	0.000
C7	0.0386 (8)	0.0464 (10)	0.0343 (9)	−0.0015 (7)	−0.0013 (7)	0.0012 (7)
C8	0.0370 (8)	0.0414 (8)	0.0335 (8)	0.0013 (6)	−0.0048 (7)	0.0050 (7)
C9	0.0377 (8)	0.0433 (9)	0.0342 (9)	0.0009 (6)	−0.0013 (7)	−0.0010 (7)
C10	0.0416 (9)	0.0457 (9)	0.0380 (9)	0.0016 (7)	0.0002 (8)	0.0064 (7)
C11	0.0541 (10)	0.0381 (9)	0.0457 (11)	0.0020 (7)	0.0009 (8)	0.0097 (7)
C12	0.0506 (10)	0.0417 (9)	0.0442 (10)	0.0015 (7)	0.0005 (8)	−0.0036 (8)
C13	0.0770 (15)	0.0650 (13)	0.0452 (13)	0.0061 (11)	0.0006 (10)	0.0205 (10)

### Geometric parameters (Å, °)

**Table:**

N1—O2	1.205 (2)	C5—C4^i^	1.409 (3)
N1—O3	1.207 (2)	C5—C6	1.441 (3)
N1—C9	1.457 (2)	C6—C1^i^	1.438 (2)
O4—C10	1.351 (2)	C7—C12	1.386 (3)
O4—C13	1.428 (3)	C7—C8	1.390 (2)
C1—C2	1.371 (3)	C8—C9	1.384 (2)
C1—C6	1.438 (2)	C8—H8	0.9300
C1—C7	1.499 (2)	C9—C10	1.396 (2)
C2—C3	1.402 (3)	C10—C11	1.391 (3)
C2—H2	0.9300	C11—C12	1.376 (3)
C3—C4^i^	1.357 (3)	C11—H11	0.9300
C3—H3	0.9300	C12—H12	0.9300
C4—C3^i^	1.357 (3)	C13—H13A	0.9600
C4—C5	1.409 (3)	C13—H13B	0.9600
C4—H4	0.9300	C13—H13C	0.9600
			
O2—N1—O3	122.34 (16)	C12—C7—C1	120.37 (16)
O2—N1—C9	117.92 (15)	C8—C7—C1	122.23 (15)
O3—N1—C9	119.67 (16)	C9—C8—C7	120.78 (15)
C10—O4—C13	117.53 (15)	C9—C8—H8	119.6
C2—C1—C6	119.70 (18)	C7—C8—H8	119.6
C2—C1—C7	115.55 (17)	C8—C9—C10	121.58 (15)
C6—C1—C7	124.75 (16)	C8—C9—N1	117.62 (14)
C1—C2—C3	122.8 (2)	C10—C9—N1	120.79 (15)
C1—C2—H2	118.6	O4—C10—C11	124.76 (15)
C3—C2—H2	118.6	O4—C10—C9	117.93 (15)
C4^i^—C3—C2	119.0 (2)	C11—C10—C9	117.31 (16)
C4^i^—C3—H3	120.5	C12—C11—C10	120.71 (17)
C2—C3—H3	120.5	C12—C11—H11	119.6
C3^i^—C4—C5	121.4 (2)	C10—C11—H11	119.6
C3^i^—C4—H4	119.3	C11—C12—C7	122.28 (17)
C5—C4—H4	119.3	C11—C12—H12	118.9
C4—C5—C4^i^	119.5 (3)	C7—C12—H12	118.9
C4—C5—C6	120.27 (13)	O4—C13—H13A	109.5
C4^i^—C5—C6	120.27 (13)	O4—C13—H13B	109.5
C1^i^—C6—C1	126.3 (2)	H13A—C13—H13B	109.5
C1^i^—C6—C5	116.87 (11)	O4—C13—H13C	109.5
C1—C6—C5	116.87 (11)	H13A—C13—H13C	109.5
C12—C7—C8	117.29 (15)	H13B—C13—H13C	109.5
			
C6—C1—C2—C3	−1.2 (3)	C1—C7—C8—C9	178.49 (14)
C7—C1—C2—C3	178.96 (19)	C7—C8—C9—C10	−0.6 (2)
C1—C2—C3—C4^i^	−0.7 (3)	C7—C8—C9—N1	−179.13 (15)
C3^i^—C4—C5—C4^i^	179.3 (2)	O2—N1—C9—C8	34.9 (2)
C3^i^—C4—C5—C6	−0.7 (2)	O3—N1—C9—C8	−142.0 (2)
C2—C1—C6—C1^i^	−177.96 (18)	O2—N1—C9—C10	−143.71 (18)
C7—C1—C6—C1^i^	1.86 (12)	O3—N1—C9—C10	39.4 (3)
C2—C1—C6—C5	2.04 (18)	C13—O4—C10—C11	0.9 (3)
C7—C1—C6—C5	−178.14 (12)	C13—O4—C10—C9	179.84 (18)
C4—C5—C6—C1^i^	−1.12 (13)	C8—C9—C10—O4	179.78 (15)
C4^i^—C5—C6—C1^i^	178.88 (13)	N1—C9—C10—O4	−1.7 (2)
C4—C5—C6—C1	178.88 (13)	C8—C9—C10—C11	−1.2 (2)
C4^i^—C5—C6—C1	−1.12 (13)	N1—C9—C10—C11	177.28 (16)
C2—C1—C7—C12	63.6 (2)	O4—C10—C11—C12	−179.82 (18)
C6—C1—C7—C12	−116.24 (17)	C9—C10—C11—C12	1.3 (3)
C2—C1—C7—C8	−112.50 (19)	C10—C11—C12—C7	0.5 (3)
C6—C1—C7—C8	67.7 (2)	C8—C7—C12—C11	−2.3 (3)
C12—C7—C8—C9	2.3 (2)	C1—C7—C12—C11	−178.55 (17)

Symmetry codes: (i) −*x*+2, −*y*+1, *z*.
